# In Vitro Assessment of Anti-Adipogenic and Anti-Inflammatory Properties of Black Cumin (*Nigella sativa* L.) Seeds Extract on 3T3-L1 Adipocytes and Raw264.7 Macrophages

**DOI:** 10.3390/medicina59112028

**Published:** 2023-11-17

**Authors:** Khawaja Muhammad Imran Bashir, Jong-Kyu Kim, Yoon-Seok Chun, Jae-Suk Choi, Sae-Kwang Ku

**Affiliations:** 1 Department of Seafood Science and Technology, The Institute of Marine Industry, Gyeongsang National University, Tongyeong 53064, Republic of Korea; imran.bashir@lstme.org; 2 German Engineering Research and Development Center for Life Science Technologies in Medicine and Environment, Busan 46742, Republic of Korea; 3 AriBnC Ltd., Yongin 16985, Republic of Korea; 4 Department of Anatomy and Histology, College of Korean Medicine, Daegu Haany University, Gyeongsan 38610, Republic of Korea

**Keywords:** pro-inflammatory mediators, Raw264.7 cells, 3T3-L1 cells, oil red O, adipogenic differentiation

## Abstract

*Background and Objectives*: This study evaluated the in vitro anti-adipogenic and anti-inflammatory properties of black cumin (*Nigella sativa* L.) seed extract (BCS extract) as a potential candidate for developing herbal formulations targeting metabolic disorders. *Materials and Methods:* We evaluated the BCS extract by assessing its 2,2-diphenyl-1-picrohydrazyl (DPPH) radical scavenging activity, levels of prostaglandin E_2_ (PGE_2_) and nitric oxide (NO), and mRNA expression levels of key pro-inflammatory mediators. We also quantified the phosphorylation of nuclear factor kappa light chain enhancer of activated B cells (NF-κB) and mitogen-activated protein kinases (MAPK) signaling molecules. To assess anti-adipogenic effects, we used differentiated 3T3-L1 cells and BCS extract in doses from 10 to 100 μg/mL. We also determined mRNA levels of key adipogenic genes, including peroxisome proliferator-activated receptor γ (PPARγ), CCAAT/enhancer binding protein α (C/BEPα), adipocyte protein 2 (aP2), lipoprotein lipase (LPL), fatty acid synthase (FAS), and sterol-regulated element-binding protein 1c (SREBP-1c) using real-time quantitative polymerase chain reaction (qPCR). *Results:* This study showed a concentration-dependent DPPH radical scavenging activity and no toxicity at concentrations up to 30 μg/mL in Raw264.7 cells. BCS extract showed an IC_50_ of 328.77 ± 20.52 μg/mL. Notably, pre-treatment with BCS extract (30 μg/mL) significantly enhanced cell viability in lipopolysaccharide (LPS)-treated Raw264.7 cells. BCS extract treatment effectively inhibited LPS-induced production of PGE_2_ and NO, as well as the expression of monocyte chemoattractant protein-1 (MCP-1), tumor necrosis factor-α (TNF-α), cyclooxygenase-2 (COX-2), inducible NO synthase (iNOS), interleukin (IL)-1β and IL-6, possibly by limiting the phosphorylation of p38, p65, inhibitory κBα (I-κBα), and c-Jun N-terminal kinase (JNK). It also significantly attenuated lipid accumulation and key adipogenic genes in 3T3-L1 cells. *Conclusions:* This study highlights the in vitro anti-adipogenic and anti-inflammatory potential of BCS extract, underscoring its potential as a promising candidate for managing metabolic disorders.

## 1. Introduction

Obesity serves as a primary contributor to a range of life-threatening metabolic conditions, including cardiovascular diseases, high blood pressure, and type 2 diabetes [1]. When obesity is present, adipocytes not only act as long-term energy storage units for lipids but also release various adipokines that play a complex role in affecting both adipocytes and non-adipocytes. This release sets the stage for prolonged inflammation and the development of associated metabolic issues, such as insulin resistance [2]. In recent years, there has been a global increase in the prevalence of metabolic syndrome, often closely linked to type 2 diabetes, due to the combined factors of obesity, a sedentary lifestyle, and high-calorie diets [3]. Aberrant fat accumulation, particularly abdominal obesity, contributes to a host of factors capable of inciting arterial arthropathy, encompassing hypertension, hyperlipidemia, coagulation abnormalities, vascular inflammation, and the irregular secretion of insulin, ultimately leading to insulin resistance [4]. Notably, obesity also serves as a driving factor in the increased incidence of fatal cardiovascular diseases [5]. Within the complex web of metabolic syndrome etiology, escalated adipocyte differentiation and chronic inflammation have emerged as pivotal factors [6]. Although there has been a proliferation of therapeutic agents for addressing metabolic syndrome, their use is constrained by a wide array of associated side effects [7]. As a result, there is a growing emphasis on actively seeking natural product-derived alternatives that offer minimal side effects [8,9,10].

Among the diverse array of natural products, black cumin (*Nigella sativa* L.) seeds [11] belonging to the Ranunculaceae family have historically found application as a spice [12]. In Islamic culture, they bear the epithet “El Habba Saouda (seeds of blessing)” and are regarded as a remedy for nearly all ailments except death [12]. Experimentally, black cumin has demonstrated antioxidant [12,13,14], anti-inflammatory [15], immunomodulatory [15,16], anti-cancer [16,17], antibacterial [18,19], antifungal [20], and hypersensitivity-inhibiting [21] effects. Black cumin houses a rich spectrum of active compounds, including phenolic compounds, like thermoquinol glucoside and coumaroyl acid derivative, flavonoids, such as apigenin and quercetin, saponins comprising triterpene saponin and alpha-hederin, and alkaloids, like nigellimine and norargemonine [22]. Flavonoids extracted from black cumin seeds have been recognized as potent protease inhibitors, including serine proteases [22]. Notably, black cumin has piqued interest as a potential agent for regulating blood sugar due to its inhibitory effects on glycated pigments [23], positioning it as a promising candidate for diabetes prevention and therapy [24,25]. A significant study by Bashir et al. [26] showcased that oral administration of black cumin seeds extract (BCS extract) at concentrations ranging from 400 to 100 mg/kg in a mild type 2 diabetes mouse model fed a high-fat diet (HFD) led to improvements across various metabolic disorders, including obesity and diabetes. These improvements likely stemmed from the regulation of glucose metabolism enzyme activity in liver tissue, concurrent activation of mitogen-activated protein kinases (MAPK), enhancement of blood lipid profiles, augmentation of antioxidant capacity, and regulation of pancreatic lipid digestion-related factors. Particularly, the administration of BCS extract (200 mg/kg) exhibited a metabolic syndrome-improving effect on par with that of metformin (250 mg/kg), a standard control drug [26]. However, investigations into the molecular mechanisms underpinning the anti-inflammatory and anti-adipogenic properties of BCS extract have been relatively limited.

Therefore, this study constitutes a pivotal step in the pursuit of functional foods designed to ameliorate metabolic diseases. We have undertaken an investigation into the in vitro anti-inflammatory and adipocyte differentiation-regulating efficacy of BCS extract, accompanied by an exploration of the associated molecular mechanisms. We evaluated the antioxidant potential of BCS extract through 2,2-diphenyl-1-picrohydrazyl (DPPH) radical scavenging assay and scrutinized its anti-inflammatory effects. Raw264.7 cells were pre-treated with BCS extract at doses ranging from 3 to 30 μg/mL, followed by exposure to 0.3 mg/mL of lipopolysaccharide (LPS), while dexamethasone (DEXA) served as a positive control drug. Within these cells, we quantified prostaglandin E_2_ (PGE_2_) and nitric oxide (NO) levels in the cell culture medium and assessed the expression levels of pro-inflammatory mediators through enzyme-linked immunosorbent assay (ELISA) and real-time quantitative polymerase chain reaction (qPCR). Additionally, we probed the impact of BCS extract on the phosphorylation of nuclear factor kappa light chain enhancer of activated B cells (NF-κB) and MAPK signaling molecules, which are activated by LPS, via immunochemical analysis. Furthermore, in order to unravel the efficacy of BCS extract with regard to the regulation of adipocyte differentiation, we induced the differentiation of murine pre-adipocyte cell line (3T3-L1) pre-adipocytes into adipocytes in the presence of BCS extract at doses ranging from 10 to 100 mg/mL, with all-*trans* retinoic acid (RA) employed as a positive control drug. Subsequent to an assessment of cytotoxicity associated with BCS extract in differentiated cells, we evaluated intracellular fat accumulation via oil red O staining and explored the mRNA expression levels of key adipogenic genes, including peroxisome proliferator-activated receptor γ (PPARγ), CCAAT/enhancer binding protein α (C/BEPα), adipocyte protein 2 (aP2), lipoprotein lipase (LPL), fatty acid synthase (FAS), and sterol-regulated element-binding protein 1c (SREBP-1c) through qPCR analysis.

## 2. Materials and Methods

### 2.1. Test Substance

A standardized BCS extract powder was generously provided by AriBnC, Yongin, Republic of Korea. The BCS extract was prepared as reported previously by Choi et al. [27] and Bashir et al. [26]. Briefly, 500 mg of BCS extract was meticulously dissolved in 10 mL of dimethyl sulfoxide (DMSO) and subjected to sterilization using a 0.2 μm syringe filter. Control drugs, namely DEXA and RA, were procured from Sigma-Aldrich, St. Louis, MO, USA, and dissolved in DMSO at a dosage of 10 mM. All test materials were stored at −20 °C for later use.

### 2.2. HPLC Analyses

The concentration of thymoquinone in the BCS extract was determined using an Agilent HPLC 1100 (Agilent Technologies, Inc., Santa Clara, CA, USA), as previously described by Bashir et al. [26]. The instrument was equipped with a Capcell Pak C18 MG II column (4.6 mm × 250 mm, 5 µm; Osaka Soda Co., Ltd., Osaka, Japan). Before injection, both standard thymoquinone (obtained from Wuhan ChemNorm Biotech Co., Ltd., Wuhan, China) and the BCS extract were dissolved in acetonitrile and filtered through 0.45 µm membrane filters. The column temperature was maintained at 30 °C, and thymoquinone was detected at 254 nm using a UV-visible absorbance detector (Agilent Technologies, Inc.). The mobile phase consisted of a blend of acetonitrile and distilled water in an 82:18 ratio. Each sample (at a concentration of 10 µg/mL) was injected in a volume of 10 µL at a flow rate of 0.9 mL/min, and the obtained results were subjected to quantitative analysis.

### 2.3. Measurement of DPPH Radical Scavenging Activity

The DPPH radical scavenging activity was measured following Choi et al.’s [9] method. Briefly, BCS extract was diluted to concentrations ranging from 30 to 1000 μg/mL in 20 μL of distilled water. Next, 150 μM of DPPH solution was added to each sample and allowed to react with the sample at room temperature for 30 min. The absorbance was subsequently recorded at a wavelength of 517 nm with an automated microplate reader (EnSpire^TM^ multimode plate reader, PerkinElmer, Waltham, MA, USA).

### 2.4. Cell Culture Conditions and Treatment of Drugs

A murine macrophage-derived cell line (Raw264.7 cells) and a murine pre-adipocyte cell line (3T3-L1 cells) were procured from the American Type Culture Collection (ATCC; Rockville, MD, USA). Raw264.7 cells were cultured in Dulbecco’s modified eagle’s medium (DMEM) supplemented with 50 μg/mL streptomycin, 50 units/mL penicillin, and 10% fetal bovine serum (FBS) at 37 °C and 5% CO_2_. As for 3T3-L1 cells, they were sub-cultured utilizing DMEM medium supplemented with 10% fetal calf serum (FCS) in place of FBS. To examine the anti-inflammatory effects of BCS extract, Raw264.7 cells were pre-treated with 3–30 μg/mL of BCS extract or 10 μM of DEXA for 30 min, followed by exposure with 0.3 mg/mL LPS for periods ranging from 0.5 to 18 h. The culture medium and cells from the treated cells were collected, stored at −20 °C, and reserved for research. To explore BCS extract’s efficacy in regulating adipocyte differentiation, 3T3-L1 pre-adipocytes were subjected to differentiation for 6 days utilizing a medium comprising 10% FBS, 1 μg/mL insulin, 1 μM DEXA, and 0.5 μM 3-isobutyl-1-methylxanthine (IBMX), in accordance with Rubin et al.’s [28] procedure. BCS extract (10–100 μg/mL) and RA (10 μM) were introduced in a similar manner [29].

### 2.5. Cell Viability Assay

The Raw264.7 cells were treated with BCS extract at concentrations ranging from 3 to 100 mg/mL for 18 h, and their viability was assessed by MTT assay, following the manufacturer’s instructions. To evaluate BCS extract’s effectiveness against LPS-induced cytotoxicity, cells were pre-treated with BCS extract at concentrations ranging from 3 to 30 μg/mL or 10 μM of DEXA for 0.5 h, followed by exposure to 0.3 μg/mL LPS for 18 h. The 10 μM concentration of dexamethasone was chosen based on the report of Chuang et al. [30]. Additionally, to verify the impact of BCS extract on 3T3-L1 cells, concentrations of 10–100 mg/mL of BCS extract or 10 μM of RA [29] were incorporated into the methylisobutylxanthine, dexamethasone, and insulin (MDI) differentiation medium throughout the differentiation process of 3T3-L1. Following the completion of treatment, living cells were subjected to 0.1 mg/mL of 2,5-diphenyl-2H-tetrazolium bromide (MTT) for 4 h. After the complete removal of the medium, the formazan crystals generated within the cells were dissolved with DMSO, and the absorbance was gauged at 570 nm.

### 2.6. Nitric Oxide Assay

NO activity was measured by Griess assay following the previously established protocols [31,32]. Raw264.7 cells were pre-treated with 3–30 μg/mL of BCS extract or 10 μM of DEXA for 0.5 h, followed by exposure with 0.3 μg/mL LPS for 18 h. The Griess reagent was then supplemented to the recovered culture medium in equivalent amounts, and the quantity of NO produced was assessed at 540 nm with an automated microplate reader (EnSpireTM).

### 2.7. ELISA Assay

The levels of PGE_2_ and tumor necrosis factor-α (TNF-α) in cell culture media were measured using the PGE_2_ assay kit (KGE004B; R&D Systems, Minneapolis, MN, USA) and the TNF-α mouse uncoated ELISA kit (88-7324-88; Invitrogen, Carlsbad, CA, USA), following the manufacturer’s instructions. Briefly, a Maxisorp 96 well microplate was coated with TNF-α-captured antibody, which was then exposed to the cell culture medium and streptavidin-conjugated horseradish peroxidase detection antibody. As for PGE_2_ content, a 96-well plate pre-coated with goat anti-mouse PGE_2_ antibody was incubated with the cell culture medium and primary antibody, followed by further interaction with horseradish peroxidase-conjugated detection PGE_2_ antibody. After a series of washes with phosphate-buffered saline (PBS) containing 0.05% Tween 20, the wells were subjected to a reaction with 3,3’,5,5’-tetramethylbenzidine (TMB; Thermo Fisher Scientific Rockford, IL, USA). The reaction was halted using a sulfuric acid solution, and the absorbance was recorded at 450 nm.

### 2.8. RNA Isolation and qPCR Analysis

Total RNA from the treated cells was isolated using Trizol solution (Invitrogen, Carlsbad, CA, USA) and quantified using a NanoDrop (ThermoFisher Scientific, Waltham, MA, USA). Subsequently, 2 μg of RNA was reverse-transcribed into cDNA using oligo-d(T)16 primers, Accupower^®^ RT PreMix (Bioneer, Daejeon, Republic of Korea), and a SimpliAmp^TM^ thermal cycler (Applied Biosystems, Waltham, MA, USA). The qPCR was conducted with gene-specific primers targeting iNOS, COX-2, PPARγ, C/BEPα, aP2, LPL, FAS, and monocyte chemoattractant protein-1 (MCP-1), SREBP-1c, TNF-α, IL-6, IL-1β (Table S1) using the diluted cDNA (50 ng), SyBr Green Ex Taq (Takara, Shiga, Japan), and the QuantStudio 5 real-time PCR instrument (Applied Biosystems, Waltham, MA, USA). To determine relative gene expression levels, we followed the method outlined by Schittgen and Livak [33]. After 40 cycles of the PCR reaction, the specificity of the reaction was confirmed through melting curve analysis, and the gene expressions were quantitated by comparing the CT values with those of the standard glyceraldehyde 3-phosphate dehydrogenase (GAPDH) gene [26].

### 2.9. Immunochemical Analysis

Immunoblotting was conducted following established protocols [31,32]. Briefly, treated cells were lysed for 1 h on ice by adding radioimmunoassay precipitation buffer containing Xpert Duo Inhibitor Cocktail Solution (GenDEPOT, Barker, TX, USA). After centrifugation (Combi 514R; Hanil Science, Daejeon, Republic of Korea) at 15,000× *g* for 10 min, supernatants were collected and used as whole cell lysates. The protein concentration in whole-cell lysates was quantified using a Bicinchoninic acid (BCA) Protein Assay Kit (23225; Thermo Fisher Scientific, Rockford, IL, USA). Equal amounts of whole-cell lysates (20 µg) were separated using 10% sodium dodecyl sulfate-polyacrylamide gels and wet transferred onto nitrocellulose membranes (GE Healthcare Life Sciences, Buckinghamshire, UK). Following the transfer, the membrane was blocked with 5% skimmed milk (Difco Laboratories, Detroit, MI, USA) and subsequently incubated with a diluted primary antibody (1:1000) followed by a horseradish peroxidase-conjugated secondary antibody (1:5000; Cat. No.: 7074 and 7076). Primary antibodies used for the detection of phosphorylated-I-κBα (Cat. No.: 2859), JNK (Cat. No.: 9252), phosphorylated-JNK (Cat. No.: 9251), phosphorylated-p65 (Cat. No.: 3033), p38 MAPK (Cat. No.: 8690), phosphorylated-p38 MAPK (Cat. No.: 9211), ERK (Cat. No.: 4695), and phosphorylated-ERK (Cat. No.: 9101) were sourced from Cell Signaling Technology (Beverly, CA, USA). Anti-p65 (Cat. No.: sc-372) and anti-β-actin (Cat. No.: A5316) antibodies were obtained from Santa Cruz Biotechnology (Santa Cruz, CA, USA) and Sigma-Aldrich (St. Louis, MO, USA), respectively. Immunoreactive proteins of interest were visualized using a West-Q Pico ECL Solution (GenDEPOT; Barker, TX, USA) with Fusion FX7 (Vilber Lourmat, Marne-la-Vallée, France). Protein expression levels were quantified using ImageJ bundled with 64-bit Java 8.

### 2.10. Oil Red O Staining

The differentiated 3T3-L1 cells were initially rinsed with PBS and then fixed with 10% formalin for 1 h. Subsequently, the cells were stained with a solution containing 60% isopropanol and 0.2% oil red O for 1 h, following the method of Suryawan and He [34]. Images of the stained cells were taken with an inverted microscope (CKX53; Olympus, Tokyo, Japan) equipped with a digital camera (Digiretina 16; Tucsen Photonics, Fuzhou, China). The oil red O dye was then dissolved in a 100% isopropanol solution, and the absorbance was recorded at a wavelength of 500 nm.

### 2.11. Statistical Analysis

All data are presented as means ± standard deviation (S.D.) of experiments conducted independently at least three times. Group means were compared using analysis of variance (ANOVA) or Welch’s method, with subsequent Levene’s test for assessing variance homogeneity [35], depending on the assumption of equal variance. Post hoc tests, such as Dunnett’s T3 or Tukey’s HSD, were employed to assess the significance of mean differences between each group.

## 3. Results

### 3.1. Detection of Thymoquinone in BCS Extract

**Figure 1:**
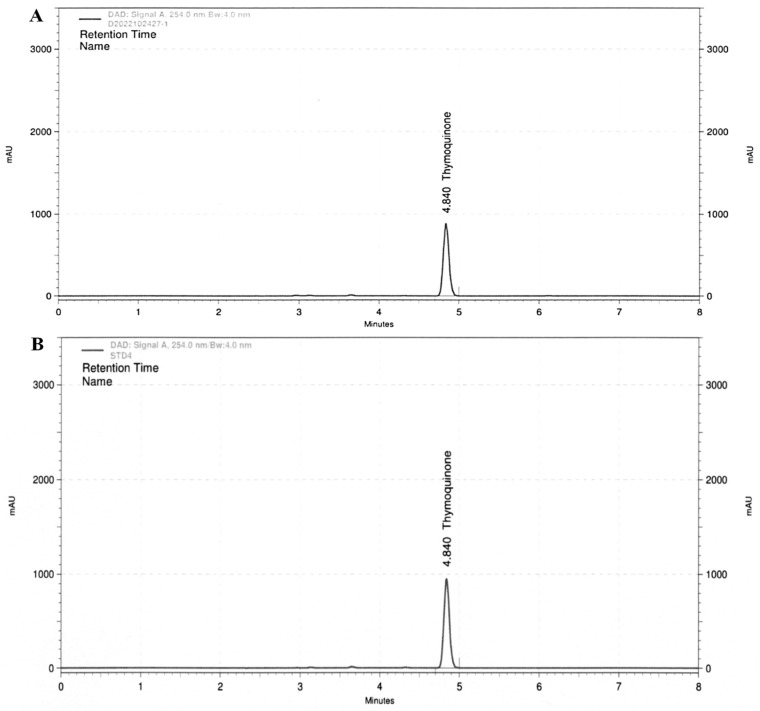
HPLC analysis of thymoquinone in the BSC extract (**A**) and standard thymoquinone (**B**).

In the HPLC analysis, both standard thymoquinone and the BCS extract exhibited a retention time of 4.840 min. This matching retention time indicates that the same chemical component, thymoquinone, is present in both the standard and the BCS extract samples. The thymoquinone concentration in the BCS extract was calculated to be 52.77 mg/g, which corresponds to approximately 5.3% of the total content. This quantification was confirmed through the HPLC analyses, as depicted in Figure 1. Thymoquinone is a bioactive compound found in black cumin seeds and is known for its potential health benefits and various pharmacological properties.

### 3.2. Free Radical Scavenging Activity Analysis

**Figure 2:**
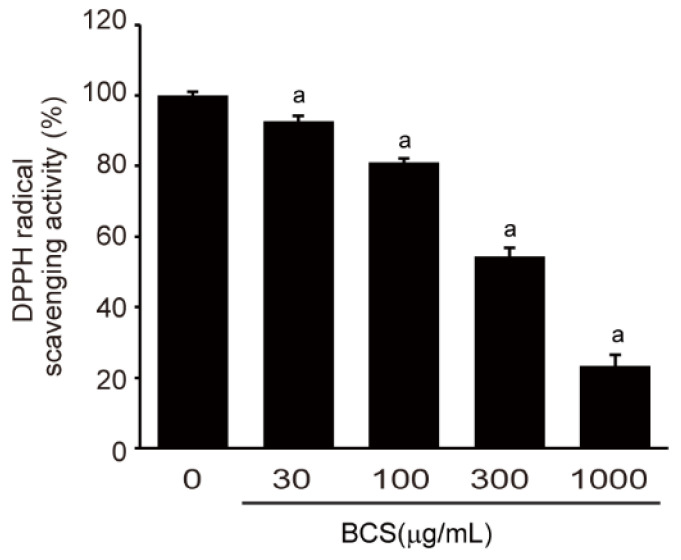
DPPH radical scavenging activity of BCS extract. DPPH-radical: 2,2-diphenyl-1-picrohydrazyl radical; BCS extract: Black cumin seeds extract. ^a^ *p* < 0.01: Significant as compared to control.

We investigated the in vitro antioxidant activity of BCS extract, as antioxidant activity plays a significant role in mitigating metabolic diseases induced by HFD. To evaluate this antioxidant potential, the DPPH radical scavenging activity assay was conducted. The BCS extract exhibited a concentration-dependent radical scavenging activity against DPPH radicals. BCS doses ranging from 30 to 1000 μg/mL showed statistically significant scavenging activity of DPPH radical. The concentration-dependent response is presented in Figure 2.

### 3.3. Anti-Inflammatory Activity of BCS Extract

#### 3.3.1. Viability of Raw264.7 Cells

**Figure 3:**
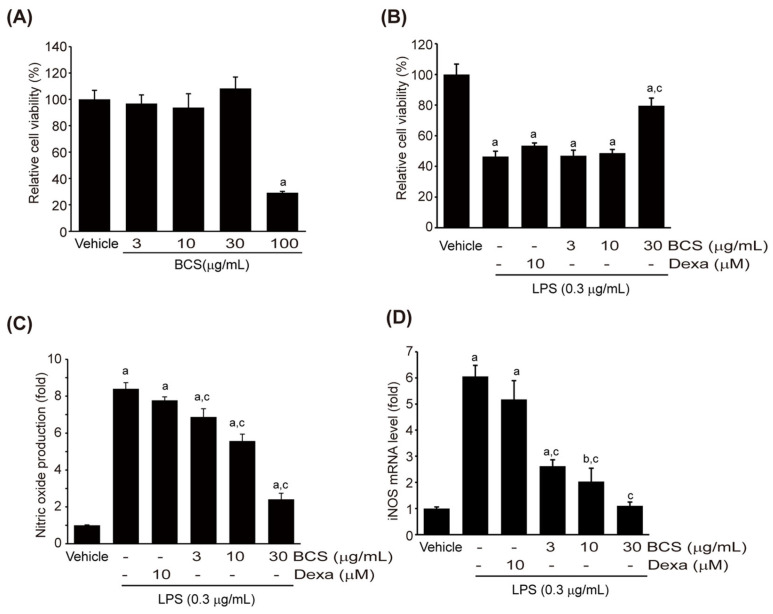
BCS extract-mediated inhibition in NO production in LPS-stimulated Raw264.7 cells. (**A**) Effect of BCS extract (3–100 μg/mL) on LPS-mediated cytotoxicity of Raw264.7 cells, (**B**) Effect of DEXA, a reference drug, on cell cytotoxicity, (**C**) NO production, and (**D**) mRNA expression of iNOS quantified by qPCR analysis. BCS extract: Black cumin seeds extract; NO: Nitric oxide; DEXA: Dexamethasone; LPS: Lipopolysaccharide; iNOS: Inducible nitric oxide synthase. ^a^ *p* < 0.01 and ^b^ *p* < 0.05: Significant as compared to control; ^c^ *p* < 0.01: Significant as compared to LPS.

To confirm cytotoxicity, Raw264.7 cells were exposed to varying concentrations of BCS (3–100 μg/mL) for 18 h, and cell viability was estimated. Treatment with BCS extract at concentrations of 3–100 μg/mL did not significantly change cell viability. However, treatment with 100 μg/mL of BCS extract led to a significant reduction in cell viability (Figure 3A). The viability percentages of Raw264.7 cells treated with 3, 10, 30, and 100 μg/mL BCS extract were 96.78 ± 6.54, 93.65 ± 10.58, 108.22 ± 8.71, and 29.22 ± 1.02% of control cells, respectively. Raw264.7 cells were pre-treated with BCS extract (3–30 μg/mL) or DEXA (10 μM) for 0.5 h, followed by treatment with 0.3 μg/mL LPS for 18 h, then cell viability was analyzed via MTT assay. LPS treatment alone significantly reduced cell viability by 46.39 ± 1.83% compared to control cells. Pre-treatment with DEXA did not mitigate the decrease in cell viability caused by LPS. However, pre-treatment with 30 μg/mL BCS extract significantly increased cell viability compared to the LPS-treated group (Figure 3B). The cell protection effects of 30 μg/mL BCS extract were even superior to that of DEXA.

#### 3.3.2. LPS-Induced NO Production

To evaluate the effects of BCS extract on LPS-induced NO production, Raw264.7 cells were pre-treated with BCS extract (3–30 μg/mL) or DEXA (10 μM) for 0.5 h and then exposed to 0.3 μg/mL LPS for 18 h. Griess reagent was reacted with the recovered cell culture medium to analyze NO production. LPS significantly increased NO production compared to control cells. Although DEXA pre-treatment did not inhibit the increase in NO production induced by LPS, pre-treatment with BCS extract led to a concentration-dependent suppression of NO production (Figure 3C). NO production levels for different treatments LPS + DEXA, LPS + 3 μg/mL BCS extract, LPS + 10 μg/mL BCS extract, and LPS + 30 μg/mL BCS extract were 8.40 ± 0.34, 7.78 ± 0.19, 6.88 ± 0.44, 5.57 ± 0.38, and 2.41 ± 0.33 fold, respectively. To investigate whether the inhibition of NO production by BCS extract pre-treatment is associated with transcriptional regulation of the iNOS enzyme, qPCR analysis was conducted to assess iNOS mRNA expression. Consistent with the results of NO, DEXA pre-treatment did not have any significant impact on LPS-induced iNOS mRNA expression. In contrast, pre-treatment with BCS extract at concentrations of 3–30 μg/mL led to a significant reduction in iNOS mRNA expression (Figure 3D).

#### 3.3.3. LPS-Induced PGE_2_ Production

**Figure 4:**
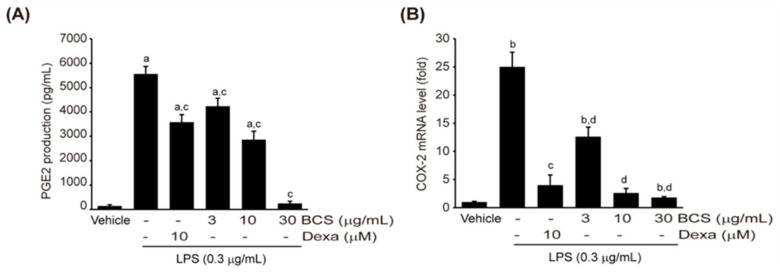
BCS extract-mediated decrease in PGE_2_ production in LPS-stimulated Raw264.7 cells. (**A**) PGE_2_ production in BCS extract (3–30 μg/mL) pre-treated Raw264.7 cells, (**B**) mRNA expression of COX-2 mRNA quantified by qPCR. BCS extract: Black cumin seeds extract; PGE2: Prostaglandin E2; COX-2: Cyclooxygenase 2; DEXA: Dexamethasone; LPS: Lipopolysaccharide. ^a^ *p* < 0.01 and ^b^ *p* < 0.05: Significant as compared to control; ^c^ *p* < 0.01 and ^d^ *p* < 0.05: Significant as compared to LPS.

PGE_2_ is an inflammatory lipid mediator produced from arachidonic acid by rate-limiting enzyme—COX [36]. Raw264.7 cells were pre-treated with various doses of BCS extract (3–30 μg/mL) or DEXA (10 μM) for 0.5 h, followed by 0.3 μg/mL LPS treatment for 18 h. The PGE_2_ content in the cell culture medium was analyzed by ELISA. LPS significantly increased the production of PGE_2_ compared to control cells. However, pre-treatment with BCS extract suppressed PGE_2_ production in a concentration-dependent manner. Furthermore, the reduction in PGE_2_ production by pre-treatment with DEXA (10 μM) was statistically significant (Figure 4A). PGE_2_ production levels by treatment with control cells, LPS, LPS + DEXA, LPS + 3 μg/mL BCS extract, LPS + 10 μg/mL BCS extract, and LPS + 30 μg/mL BCS extract were 144.40 ± 55.10, 5552.59 ± 311.67, 3581.20 ± 310.85, 4231.47 ± 326.04, 2861.11 ± 347.89, and 250.01 ± 87.46 pg/mL, respectively. To investigate whether the inhibition of PGE_2_ production by BCS extract pre-treatment is associated with the regulation of COX-2 expression, qPCR analysis was performed to assess COX-2 mRNA expression. Consistent with the results of PGE_2_ production, pre-treatment with DEXA and BCS extract significantly reduced the COX-2 mRNA expression induced by LPS stimulation. The reduction effect of 10 and 30 μg/mL BCS extract pre-treatment on COX-2 mRNA expression was equivalent to that of DEXA (Figure 4B).

#### 3.3.4. Effect of BCS Extract on LPS-Induced Pro-Inflammatory Cytokine Production

**Figure 5:**
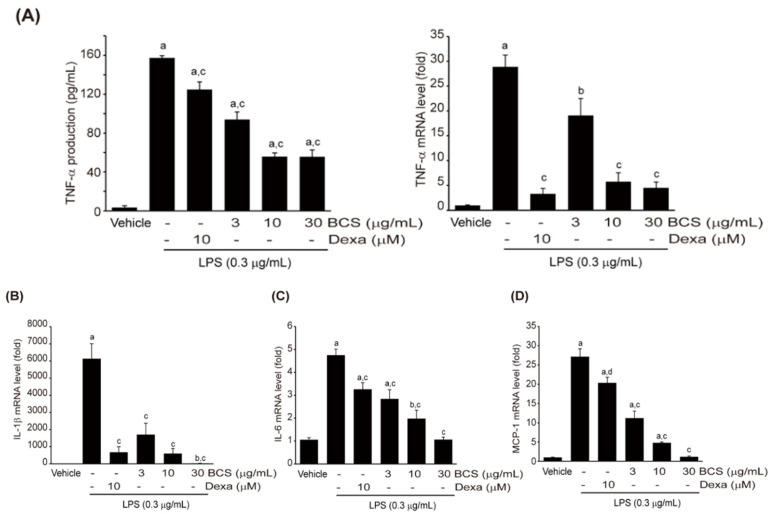
BCS extract-mediated reduction in pro-inflammatory cytokines production in LPS-stimulated Raw264.7 cells. mRNA expression levels of TNF-α (**A**), IL-1β (**B**), IL-6 (**C**), and MCP-1 (**D**) quantified by ELISA or qPCR. Raw264.7 cells were treated with BCS extract (3–30 μg/mL) for 0.5 h and then LPS (0.3 μg/mL) for 6 h (**A**-**right**, **B**–**D**) or 18 h (**A**-**left**). BCS extract: Black cumin seeds extract; TNF-α: Tumor necrosis factor-α; DEXA: Dexamethasone; LPS: Lipopolysaccharide; IL: Interleukin; MCP-1: Monocyte chemoattractant protein 1. ^a^ *p* < 0.01 and ^b^ *p* < 0.05: Significant as compared to control; ^c^ *p* < 0.01 and ^d^ *p* < 0.05: Significant as compared to LPS.

Pro-inflammatory cytokines, including MCP-1, TNF-α, IL-1β, and IL-6, are produced by natural immune cells, including macrophages, and play a significant role in the inflammatory response [37,38]. To assess the impact of BCS extract on the production of these cytokines induced by LPS, Raw264.7 cells were pre-treated with different doses of BCS extract (3–30 μg/mL) or DEXA (10 μM) for 0.5 h. Subsequently, the cells were treated with 0.3 μg/mL LPS for 6 h (analyzed by qPCR) or 18 h (analyzed by ELISA). LPS significantly enhanced the production of MCP-1, TNF-α, IL-1β, and IL-6 compared to control cells. Pre-treatment with DEXA significantly inhibited the production of these pro-inflammatory cytokines. BCS extract pre-treatment at concentrations of 3–30 μg/mL statistically significantly suppressed the production of all measured pro-inflammatory cytokines, except for TNF-α mRNA at 3 μg/mL concentration (Figure 5A–D).

#### 3.3.5. Effect of BCS Extract on LPS-Induced MAPK and NF-κB Phosphorylation

**Figure 6:**
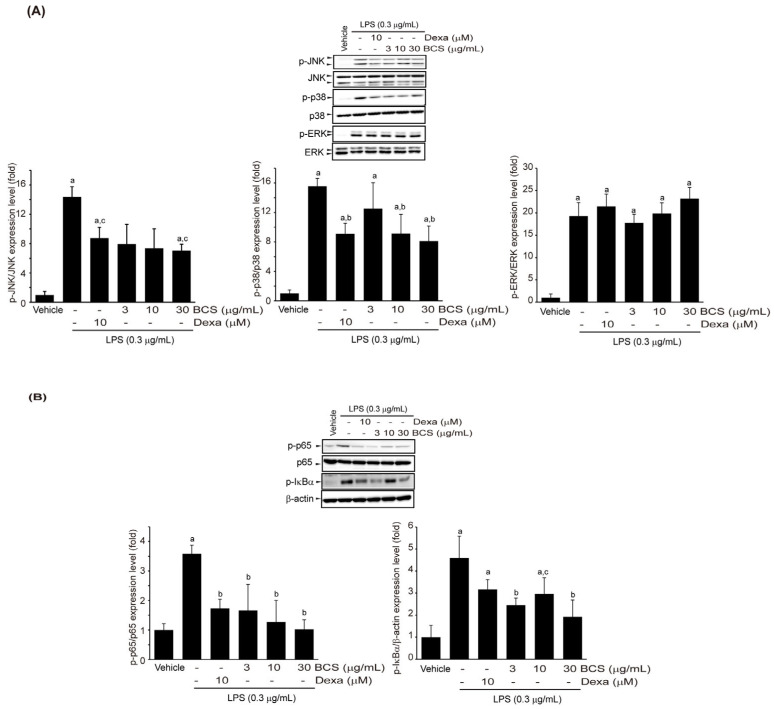
BCS extract-mediated inhibition in MAPKs and NF-κB phosphorylation in LPS-stimulated Raw264.7 cells. Raw264.7 cells were exposed to BCS extract (3–30 μg/mL) for 0.5 h and then LPS (0.3 μg/mL) for 0.5 h. Phosphorylation levels of MAPKs (**A**) and NF-κB (**B**) were quantified by immunoblotting. BCS extract: Black cumin seeds extract; JNK: c-Jun N-terminal kinase; p38 MAPK: DEXA: Dexamethasone; LPS: Lipopolysaccharide; p38 mitogen-activated protein kinase; ERK: Extracellular signal-regulated kinase; I-κBα: Inhibitory κBα; MAPK: Mitogen-activated protein kinases; NF-κB: Nuclear factor kappa-light-chain-enhancer of activated B cells. ^a^ *p* < 0.01: Significant as compared to control; ^b^ *p* < 0.01 and ^c^ *p* < 0.05: Significant as compared to LPS.

To further investigate the molecular mechanism underlying the anti-inflammatory effects of BCS extract, Raw264.7 cells were exposed to different doses of BCS extract (3–30 μg/mL) or DEXA (10 μM) for 0.5 h. The cells were then treated with 0.3 μg/mL LPS for 0.5 h. Phosphorylation of MAPK and NF-κB proteins was analyzed by immunochemical methods after electrophoresis using whole-cell protein extracts. LPS increased phosphorylation of I-κBα, JNK, p38, p65, and extracellular signal-regulated kinase (ERK). Pre-treatment with BCS extract inhibited phosphorylation of these signaling molecules, except ERK. Notably, inhibition of p38 phosphorylation by 10–30 μg/mL of BCS extract pre-treatment, JNK phosphorylation by 30 μg/mL of BCS extract pre-treatment, and phosphorylation of I-κBα and p65 by 3–30 μg/mL of BCS extract pre-treatment was significant compared to LPS treatment alone and were similar in efficacy of DEXA (Figure 6A,B).

### 3.4. Adipocyte Differentiation Regulatory Activity of BCS Extract

#### 3.4.1. Effect of BCS Extract on Lipid Accumulation in 3T3-L1 Cells

**Figure 7:**
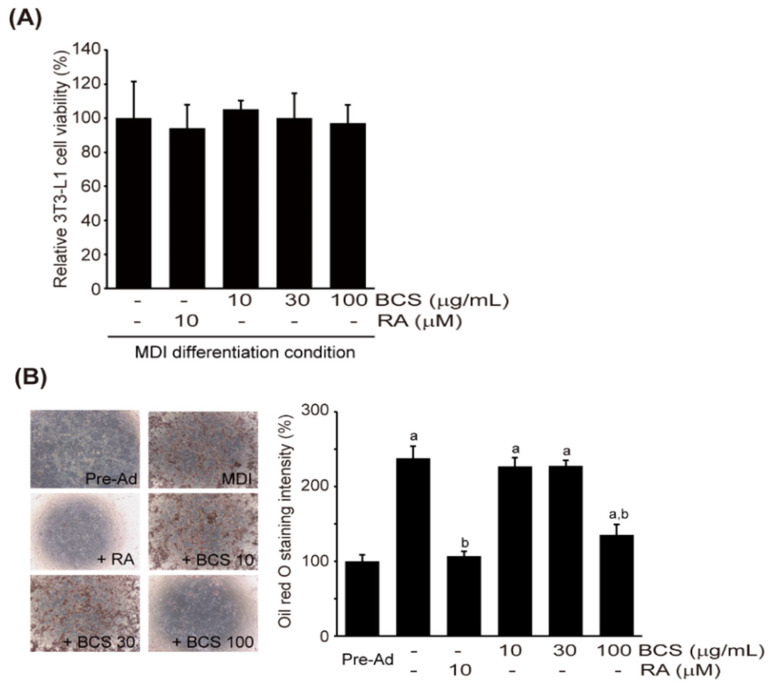
BCS extract-medicated inhibition in lipid accumulation during 3T3-L1 cell differentiation. (**A**) Cell viability of 3T3-L1 cells differentiated into adipocyte-like cells in the presence of either BCS extract (10–100 μg/mL) or RA (10 μM). (**B**) Quantification of lipid accumulation after oil red O staining. BCS extract: Black cumin seeds extract; 3T3-L1: Murine pre-adipocyte cell line; RA: All trans retinoic acid; MDI: Methylisobutylxanthine, dexamethasone, and insulin differentiation medium. ^a^ *p* < 0.01: Significant as compared to control; ^b^ *p* < 0.01: Significant as compared to MDI.

Prior to studying the impact of BCS extract on the differentiation of 3T3-L1 cells into adipocytes, the impact of BCS extract on the survival rate of 3T3-L1 cells was assessed. 3T3-L1 pre-adipocytes were induced to differentiate into adipocytes using MDI, and BCS extract at concentrations of 10–100 µM or 10 µM of RA were added to the differentiation medium. The viability results of 3T3-L1 cells revealed that treatment with 10–100 μM of BCS extract or 10 μM of RA did not significantly affect their viability (Figure 7A). Changes in cell viability by treatment with MDI + RA, MDI + 10 μg/mL BCS extract, MDI + 30 μg/mL BCS extract, and MDI + 100 μg/mL BCS extract were 94.08 ± 13.85, 105.33 ± 105.33 ± 94.08 ± 13.85, 105.33 ± 4.93, 100.00 ± 14.64, and 97.04 ± 10.76%, respectively. Fat granules in differentiated cells were stained with oil red O, and the analysis revealed that treatment with 10 μM of RA completely inhibited the accumulation of fat granules induced by MDI. Furthermore, treatment with 100 μg/mL of BCS extract also significantly reduced the production of fat granules compared to MDI-differentiated cells (Figure 7B).

#### 3.4.2. Expression of Genes Related to Adipocyte Differentiation

**Figure 8:**
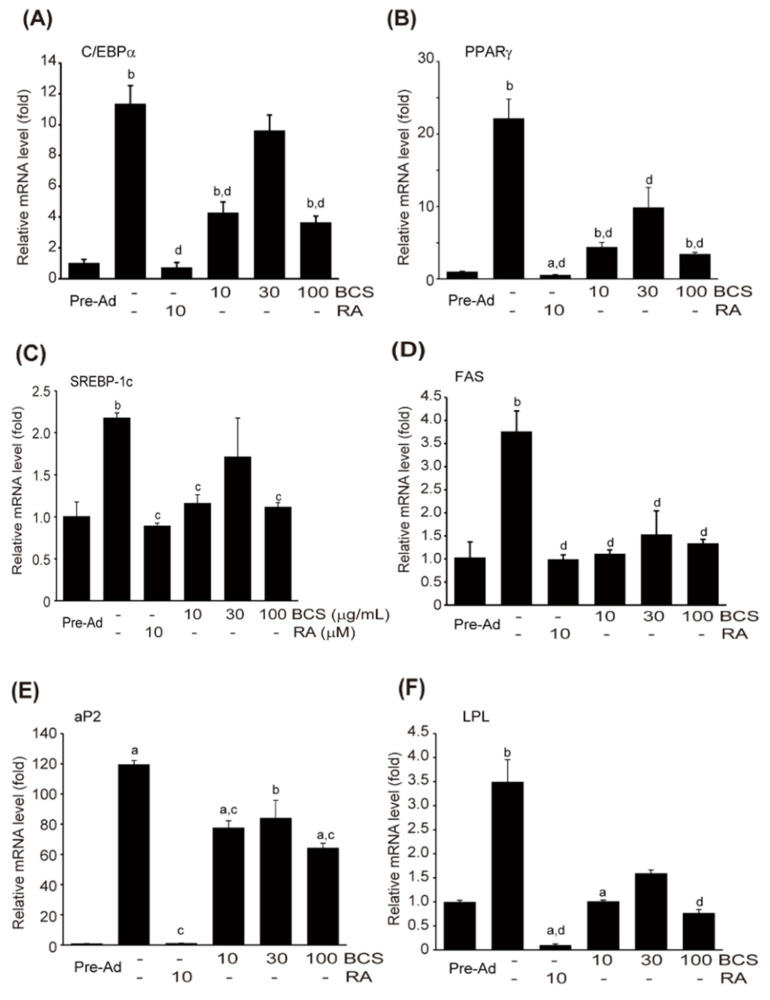
BCS extract-mediated inhibition in mRNA expression of adipogenic genes during 3T3-L1 cell differentiation. qPCR quantified mRNA expressions of C/EBPα (**A**), PPARγ (**B**), SREBP-1c (**C**), FAS (**D**), aP2 (**E**), and LPL (**F**) in 3T3-L1 differentiated into adipocyte-like cells in the presence of either BCS extract (10–100 μg/mL) or RA (10 μM). BCS extract: Black cumin seeds extract; 3T3-L1: Murine pre-adipocyte cell line; C/BEPα: CCAAT/Enhancer binding protein α; RA: All trans retinoic acid; PPARγ: Peroxisome proliferator-activated receptor γ; SREBP-1c: Sterol regulatory element binding protein 1c; aP2: Adipocyte protein 2; LPL: Lipoprotein lipase. ^a^ *p* < 0.01 and ^b^ *p* < 0.05: Significant as compared to control; ^c^ *p* < 0.01 and ^d^ *p* < 0.01: Significant as compared to MDI.

3T3-L1 cells were induced to differentiate into adipocytes using MDI and treated with 10–100 μM of BCS extract or 10 μM of RA. The impact on the expression of gene families known to affect adipocyte differentiation was assessed through qPCR. The differentiation into adipocytes using MDI led to a significant increase in the mRNA expression of PPARγ, C/BEPα, aP2, LPL, FAS, and SREBP-1c gene families, which are associated with adipocyte differentiation. However, RA treatment significantly suppressed the mRNA expression of these gene families. Treatment with BCS extract also exhibited a trend toward the suppression of these gene families. Furthermore, the reduction in the mRNA expression of SREBP-1c, C/EBPα, and aP2 induced by 10 and 100 μg/mL of BCS extract treatment, as well as the reduction in PPARγ and FAS mRNA expression induced by 10–100 μg/mL of BCS extract treatment, were statistically significant compared to MDI differentiated cells (Figure 8A–F).

## 4. Discussion

The global rise in metabolic syndrome prevalence has contributed to the increasing incidence of non-alcoholic fatty liver disease (NAFLD). NAFLD is characterized by an excessive buildup of fats in liver tissue [39]. This fat accumulation damages liver cells and leads to fibrosis and inflammation, ultimately progressing to severe conditions like hepatocellular carcinoma, liver cirrhosis, and non-alcoholic steatohepatitis [40,41]. Additionally, the increased accumulation in liver cells, including triglycerides, diglycerides, and ceramides, results in insulin resistance, which is a precursor to severe type 2 diabetes and related cardiovascular diseases [42,43]. Unfortunately, there are currently no known drugs that comprehensively treat metabolic syndrome [41]. While some drugs are employed to manage its symptoms, there is still no developed medication that addresses the metabolic syndrome as a whole [44]. Furthermore, the existing treatments often come with various side effects, limiting their usage [7]. Lifestyle adjustments, such as exercise therapy for weight loss, remain a primary approach to managing metabolic syndrome [41]. Hence, there is an urgent demand for a low-side-effect drug that can effectively alleviate metabolic syndrome, especially one that can control oxidative stress, a recognized contributor to diabetes and associated complications, alongside blood sugar regulation [9,10,45]. This has led to active research into effective antioxidants or α-glucosidase inhibitors with minimal adverse effects [9,10,46,47].

Black cumin has been experimentally proven to possess a range of health benefits, including antioxidant [12,13,14], anti-inflammatory [15], immunomodulatory [15,16], anti-cancer [16,17], antibacterial [18,19], antifungal [20], and hypersensitivity inhibition [21] effects. Notably, black cumin’s ability to control blood sugar by inhibitory glycated pigment formation has garnered attention as a potential therapeutic and preventive agent for diabetes [23,24,25]. Consequently, efforts have been made to develop functional food materials and new drugs derived from natural products, using 45% Kcal HFD-fed mice as experimental models for obesity and mild type 2 diabetes to improve obesity and diabetes-related complications [8,9,10,48]. This study investigated the dose-dependent impact of black cumin seed (BCS) extract (100–400 mg/kg) in improving complications associated with obesity and diabetes, comparing its efficacy to metformin (250 mg/kg), a standard treatment for obesity and type 2 diabetes [8,9,10]. The BCS extract administration ameliorated various metabolic disorders, including anti-diabetic and anti-obesity properties. This was achieved through the regulation of glucose metabolism enzyme activity in liver tissue, activation of MAPK, improvements in blood lipid profiles and antioxidant capacity, as well as the modulation of factors related to lipid digestion in the pancreas. While the impact of 200 mg/kg BCS extract administration was comparable to that of 250 mg/kg metformin, a control drug [26], research on the molecular mechanisms related to inflammation and fat accumulation in the BCS extract treatment group has been relatively limited. Therefore, we evaluated the anti-inflammatory and adipocyte differentiation-regulating effects of BCS extract to develop functional food for mitigating metabolic diseases.

The study was initiated by examining the antioxidant properties of BCS extract, which play a crucial impact in reducing inflammatory responses by shielding cells from lipotoxic stress. The results demonstrated statistically significant DPPH-radical scavenging activity in BCS extract treatments within the range of 30–1000 μg/mL. Furthermore, through MTT analysis, it was confirmed that pre-treatment with BCS extract at concentrations up to 30 μg/mL could mitigate the cytotoxicity induced by LPS treatment in Raw264.7 cells. Thus, BCS extract exhibited antioxidant activity capable of scavenging free radicals without inducing toxicity, at least under the conditions of this experiment.

Inflammation represents a key mechanism in the initiation of metabolic diseases [49], and previous research by our group has demonstrated that BCS extract effectively suppresses and prevents metabolic diseases induced by a high-fat diet [26]. In the current study, we delved into the anti-inflammatory impact of BCS extract in Raw264.7 cells stimulated by LPS, investigating both cellular and molecular levels to understand its impact on inflammation regulation. Nitric oxide (NO), synthesized from L-arginine by the NO synthase family [50], involves three known types: (i) inducible NOS (iNOS), (ii) neuronal NOS (nNOS), and (iii) endothelial NOS (eNOS). The expression of iNOS is induced by inflammatory stimuli, including LPS [51,52]. While physiological NO production is involved in attacking and eliminating pathogens through phagocytosis, excessive and continuous NO production can lead to tissue damage via reactive nitrogen species. Therefore, the evaluation of NO production inhibition is commonly used to identify candidate drugs with anti-inflammatory properties. Our study found that pre-treatment of Raw264.7 cells with BCS extract led to a concentration-dependent reduction in NO production and iNOS mRNA expression, simultaneously increasing cell viability reduced by LPS. This suggests that BCS extract regulates NO production by modulating the LPS-dependent signaling pathway without compromising cell viability.

Prostanoids, including thromboxanes and four prostaglandins, are icosane derivatives produced from arachidonic acid released from cell membrane phospholipids through a series of biosynthetic cycles involving COX [36]. Two COX isozymes have been reported thus far, with COX-1 primarily involved in maintaining tissue homeostasis. In contrast, COX-2 expression is typically low in normal physiological conditions but rapidly increases in various pathological conditions, particularly inflammation [53]. COX-2-derived prostanoids significantly contribute to the progression of inflammation [54]. Therefore, this study suggests that the suppression of PGE_2_ production following BCS extract treatment is recognized as the transcriptional regulation of COX-2.

MCP-1, TNF-α, IL-1β, and IL-6 are prominent pro-inflammatory cytokines produced by innate immune cells, capable of triggering various immune and inflammatory responses [37,38]. For instance, IL-1β activates natural killer cells and B/T lymphocytes and influences the hypothalamus to induce heat generation during the inflammatory process [55,56]. IL-6 not only enhances antibody production by activated lymphocytes but also plays a pivotal role in acute inflammatory responses. TNF-α is recognized as a key factor that elevates the expressions of other pro-inflammatory cytokines in the early stages of autoimmune diseases and inflammatory responses [55,57], while MCP-1 serves as a chemokine that promotes monocyte infiltration and migration into damaged areas [58]. In this study, it was demonstrated that BCS extract significantly suppressed the production of these pro-inflammatory mediators, which are typically elevated by LPS. These findings indicate that the anti-inflammatory effect of BCS extract involves the regulation of common signaling molecules associated with the production of these cytokines.

Among the various signaling molecules related to the initiation of inflammatory responses, LPS binding to toll-like receptor 4 (TLR4) triggers the activation of adapter molecules, including TIR-domain-containing adapter-inducing interferon-β (TRIF) and myeloid differentiation primary-response gene 88 (MyD88). Activated receptors facilitate the polyubiquitination of tumor necrosis factor receptor-associated factor 6 (TRAF6) and induce the phosphorylation of transforming growth factor-beta-activated kinase 1 (TAK1), which transmits intracellular signals to activate MAPK and NF-κB pathways [37,59]. In the unstimulated state, NF-κB is located in the cytoplasm, bound to I-kB; however, LPS/TLR4 stimulation leads to phosphorylation of I-κB through the activation of MyD88 and TRIF-dependent IκB kinase (IKK). Consequently, NF-κB homo/heterodimers are released from I-κB, migrate to the nucleus, and promote the expression of the aforementioned pro-inflammatory mediators [59]. Additionally, the activation of MAPKs not only stimulates the expression of inflammatory mediators via activating protein-1 (AP-1) but also regulates the NF-κB signaling pathway by facilitating the I-κB degradation [59,60]. Moreover, IKK is known as an upstream kinase that promotes the phosphorylation of the topologically associating domain (TAD region) of NF-kB. Thus, this study revealed that BCS extract inhibited the phosphorylation of MAPKs and p65 (with the exception of ERK), indicating that BCS extract can regulate the expression of inflammatory mediators by impeding the activity of NF-κB and AP-1 transcription factors.

Over the last four decades, in vitro cell systems related to adipocyte differentiation and adipogenesis have been extensively investigated [61]. Our understanding of the differentiation process at the molecular and cellular levels has improved significantly. Among various pre-adipocytes, 3T3-L1 cells, a subtype of Swiss 3T3 cells derived from day 17 and day 19 mouse embryos [62], resemble fibroblasts in morphology during the growth phase. Inducing the differentiation of 3T3-L1 cells through appropriate stimulation results in fat accumulation accompanied by changes in cell morphology. These cells naturally differentiate into adipocytes with continuous culture in FCS over several weeks, and differentiation is accelerated when cultured in FBS containing DEXA, IBMX, and insulin [28]. This study found that BCS extract, at a dosage of 100 mg/mL, could inhibit lipid accumulation in 3T3-L1 cells without causing cytotoxicity.

During the differentiation of pre-adipocytes, cell growth arrest, and clonal expansion are accompanied by changes in various genes. Notably, an increase in lipoprotein lipase (LPL) mRNA expression has been identified as a major early marker of adipocyte differentiation [63]. LPL plays a key role in regulating lipid accumulation by being secreted from mature adipocytes [64]. Moreover, in the early stages of adipocyte differentiation, at least two or more C/EBPs and PPARγ transcription factors are induced. These early inductions of C/EBPs and PPARγ contribute to the eventual differentiation into adipocytes by stimulating the expressions of adipocyte-specific genes. PPARγ, in particular, is expressed in small quantities in pre-adipocytes but gradually increases in expression during the differentiation process after two days, with peak expression occurring in mature adipocytes [65]. The transient expression of C/EBPβ/δ during adipocyte differentiation induces PPARγ expression, and reduced expression of C/EBPβ/δ in the mid-differentiation phase enhances C/EBPα expression, thereby inducing adipocyte-specific gene expression [65]. Furthermore, during adipocyte differentiation, it has been reported that SREBP-1c, a transcription factor with a basic helix–loop–helix (bHLH) leucine zipper motif, is induced in the early stages to promote fatty acid biosynthesis [66]. Toward the end of adipocyte differentiation, fat biosynthesis is activated, leading to the expression of adipocyte-specific fat-binding proteins like aP2 [67]. In this study, we found that BCS extract significantly and dose-dependently suppressed the expression of SREBP-1c, C/EBPα, and aP2 genes. Although these differences were statistically significant, the treatment with 30 µg/mL of BCS extract led to increased mRNA levels. While BCS extract appeared to regulate the activity of key transcription factors involved in adipocyte differentiation, follow-up studies are necessary to understand their mechanisms of expression. Moreover, while treatment with BCS extract at concentrations ranging from 10 to 100 μg/mL showed substantial changes in mRNA expressions of pro-inflammatory cytokines and genes related to adipocyte differentiation as analyzed by qPCR and ELISA, obtaining more reliable data may require Western blotting to detect protein signals. Specifically, for p-I-κBα, phosphorylation is of importance, and therefore, we monitored only p-I-κBα expression in this study. However, it might be beneficial to consider the expression of I-κBβ in future investigations.

The disparities in TNFα gene and protein expression may stem from various factors, including DEXA pre-treatment, TNF signaling, time intervals between protein production changes, mRNA loading volume, mRNA expression, and assay sensitivity. The quantification of these variations between mRNA signals and western blot outcomes can be achieved through densitometry and normalization, as suggested by Antras-Ferry [68]. Previous studies have indicated that DEXA, acting as a glucocorticoid, exhibits cell- or tissue-specific activity [68,69]. Notably, pre-treating with DEXA significantly hampers the production of pro-inflammatory cytokines, such as TNF-α. Consequently, the variations in TNF-α effect in this study could be elucidated by the DEXA pre-treatment of the cells under investigation. The effects of glucocorticoid hormones involve binding to intracellular glucocorticoid receptors, which then bind to regulatory elements in the 5′ flanking region of target genes [70]. Some studies propose a protein–protein interaction between the glucocorticoid receptor and the transcription factor NF-kB, indicating negative crosstalk in DEXA and TNF-α-induced gene transcription [71]. While this research suggests the involvement of the glucocorticoid receptor in DEXA effects, there is no evidence supporting crosstalk between NF-kB and the receptor. Furthermore, the impact of DEXA on gene expression may depend on TNF signaling effects [72,73]. Therefore, a comprehensive study should be conducted to better understand the impact of these factors.

The emergence of the obesity phenotype entails significant alterations in adipocytes and various cellular processes, resulting in a notable rise in systemic oxidative stress mediated by reactive oxygen species. These modifications instigate a persistent state of inflammation within the body, fueled by a redox imbalance. Chronic inflammation and systemic oxidative stress collectively play a crucial role in perpetuating obesity and exacerbating the onset of cardiovascular complications, non-alcoholic steatohepatitis, dyslipidemia, Type 2 diabetes, and other conditions where obesity is a significant risk factor. In prior investigations, we have demonstrated a direct correlation between antioxidant activity and the anti-obesity impact in various obesity models, including the high-fat diet-fed obese mouse model [9,10,26]. Mechanistically, distinct antioxidants contribute to normalizing inflammatory responses, facilitating the transition toward non-obese phenotypes [74]. Through modulation of inflammatory cascades, such as NF-kβ, antioxidants diminish the levels of various inflammatory mediators, including TNF-α, IL-6, and IL-1 β [74,75,76]. Furthermore, antioxidants reinstate the redox state during altered thermogenesis under diseased metabolic conditions [77]. Antioxidants boost the activity of differentiative and proliferative cascades within adipocytes, resulting in improved expressions of C/EBPα and PPARγ [9,10,78,79,80,81]. These effects lead to numerous enhancements, including increased insulin sensitivity, improved adipocyte functionality, reduced circulating triglycerides, adipogenesis, and decreased fat mass [74]. This suggests that each antioxidant holds the potential to mitigate the development and progression of associated comorbidities through distinct mechanisms, highlighting a robust connection between antioxidant action and the alleviation of obesity-induced oxidative damage and metabolic dysfunction. The outcomes of the present study further reinforce the notion that the antioxidant activity and anti-obesity effects of BCS extract are closely linked. However, additional research is imperative to ascertain the effectiveness of antioxidants working synergistically with each other and in conjunction with current pharmacotherapy to prevent or reverse the associated comorbidities of obesity.

## 5. Conclusions

The BCS extract exhibited promising in vitro anti-inflammatory and adipocyte differentiation-regulating effects. BCS extract showed a concentration-dependent increase in DPPH radical scavenging activity, highlighting its antioxidant potential. This property can protect cells from oxidative stress. In LPS-activated Raw264.7 cells, BCS extract significantly suppressed the production of various pro-inflammatory mediators, including MCP-1, TNF-α, PGE_2_, NO, iNOS, IL-1β, and COX-2. It also inhibited the phosphorylation of NF-κB and the activation of MAPK, both of which are critical pathways in the inflammatory response. Furthermore, BCS extract effectively inhibited fat granule production during the differentiation process of key genes associated with adipocyte differentiation, including PPARγ, C/EBPα, SREBP-1c, FAS, LPL, and aP2. These genes are important regulators of lipid metabolism and adipogenesis. These findings collectively suggest that BCS extract holds significant potential for development as a health functional food or natural drug material aimed at improving metabolic diseases. Its dual action in combating inflammation and regulating adipocyte differentiation makes it a promising candidate for further research and development in the field of metabolic syndrome and related conditions.

## Data Availability

Data are contained within the article.

## Supplementary Materials

The following supporting information can be downloaded at https://www.mdpi.com/article/10.3390/medicina59112028/s1, Table S1: Oligonucleotides used in the present study.

## Abbreviations

3T3-L1: Murine pre-adipocyte cell line; ANOVA: Analysis of variance; AP-1: Activating protein-1; aP2: Adipocyte protein 2; AS: Fatty acid synthase; ATCC: American type culture collection; BCA: Bicinchoninic acid; BCS extract: Black cumin seeds extract; bHLH: Basic helix-loop-helix; C/BEPα: CCAAT/Enhancer binding protein α; cDNA: Complementary DNA; COX-2: Cyclooxygenase 2; CT: Cycle threshold; DEXA: Dexamethasone; DMEM: Dulbecco’s modified eagle’s medium; DMSO: Dimethyl sulfoxide; DPPH-radical: 2,2-diphenyl-1-picrohydrazyl radical; DT3: Dunnett’s T3 test; ELISA: Enzyme-linked immunosorbent assay; eNOS: Endothelial nitric oxide synthase; ERK: Extracellular signal-regulated kinase; FAS: Fatty acid synthase; FBS: Fetal bovine serum; FCS: Fetal calf serum; GAPDH: Glyceraldehyde 3-phosphate dehydrogenase; HFD: High-fat diet; IBMX: 3-isobutyl-1-methylxanthine; IKK: TRIF-dependent IκB kinase; IL: Interleukin; iNOS: Inducible nitric oxide synthase; JNK: c-Jun N-terminal kinase; LPL: Lipoprotein lipase; LPS: Lipopolysaccharide; MAPK: Mitogen-activated protein kinases; MCP-1: Monocyte chemoattractant protein 1; MDI: Methylisobutylxanthine, dexamethasone, and insulin differentiation medium; MTT: 2,5-diphenyl-2H-tetrazolium bromide; MyD88: Myeloid differentiation primary-response gene 88; NF-κB: Nuclear factor kappa-light-chain-enhancer of activated B cells; nNOS: Neuronal nitric oxide synthase; NO: Nitric oxide; p38 MAPK: p38 mitogen-activated protein kinase; PBS: Phosphate buffered saline; PGE2: Prostaglandin E2; PPARγ: Peroxisome proliferator-activated receptor γ; qPCR: Real-time quantitative polymerase chain reaction; RA: All trans retinoic acid; Raw264.7: Murine macrophage-derived cell line; SDS-PAGE: Sodium dodecyl-sulfate polyacrylamide gel electrophoresis; SREBP-1c: Sterol regulatory element binding protein 1c; TAD: Topologically associating domain region; TAK1: Transforming growth factor beta-activated kinase 1; THSD: Tukey’s honest significant difference test; TLR4: Toll-like receptor 4; TMB: 3,3’,5,5’-tetramethylbenzidine; TNF-α: Tumor necrosis factor-α; TRAF6: Tumor necrosis factor receptor associated factor 6; TRIF: TIR-domain-containing adapter-inducing interferon-β; κBα; Inhibitory κBα.
