# Supplementary material for: In Vitro Assessment of Anti-Adipogenic and Anti-Inflammatory Properties of Black Cumin (Nigella sativa L.) Seeds Extract on 3T3-L1 Adipocytes and Raw264.7 Macrophages
Source: Medicina (Kaunas). 2023 Nov 17;59(11):2028. doi: 10.3390/medicina59112028 (PMC10673321; doi:10.3390/medicina59112028)
Supplement: Supplementary file 1 [file medicina-59-02028-s001.zip › medicina-2638102-supplementary.pdf]

medicina

MDPI

Article

In Vitro Assessment of Anti-Adipogenic and Anti-Inflammatory Properties of Black Cumin (*Nigella sativa* L.) Seeds Extract on 3T3-L1 Adipocytes and Raw264.7 Macrophages

Khawaja Muhammad Imran Bashir [1,2], Jong-Kyu Kim [3], Yoon-Seok Chun [3], Jae-Suk Choi [1,*], Sae-Kwang Ku [4,*]

1 Department of Seafood Science and Technology, The Institute of Marine Industry, Gyeongsang National University, Tongyeong-si, Gyeongsangnam-do 53064, Korea; imran.bashir@lstme.org
2 German Engineering Research and Development Center for Life Science Technologies in Medicine and Environment, Busan 46742, Korea
3 AriBnC, Ltd., Yongin-si, Gyeonggi-do 16985, Korea
4 Department of Anatomy and Histology, College of Korean Medicine, Daegu Haany University, Gyeongsan 38610, Korea
* Correspondence: jsc1008@gnu.ac.kr (J.-S.C.); gucci200@dhu.ac.kr (S.-K.K.); Tel.: +82-55-772-9142 (J.-S.C.); +82-53-819-1549 (S.-K.K.)

Table S1. Oligonucleotide sequences used in this study.

Gene Symbol	Forward Sequence (5′ – 3′)	Backward Sequence (5′ – 3′)	Genbank Accesion No.	Annealing Temp. (ºC)	Product size (bp)
aP2	CATGGCCAAGCCCAACAT	CGCCCAGTTTGAAGGAAATC	NM_024406.4	65	101
C/EBPα	AGCAACGAGTACCGGGTACG	TGTTTGGCTTTATCTCGGCTC	NM_007678.3	65	71
FAS	CTGAGATCCCAGCACTTCTTGA	GCCTCCGAAGCCAAATGAG	NM_007988.3	65	101
LPL	GGCCAGATTCATCAACTGGAT	GCTCCAAGGCTGTACCCTAAG	NM_008509.2	65	81
PPARγ	CGCTGATGCACTGCCTATGA	AGAGGTCCACAGAGCTGATTCC	NM_011144.6	65	101
SREBP-1c	GGAGCCATGGATTGCACATT	GCTTCCAGAGAGGAGGCCAG	NM_011480.3	65	182
iNOS	CCTCCTCCACCCTACCAAGT	CACCCAAAGTGCTTCAGTCA	NM_010927.3	56	199
COX-2	TCCAGATCACATTTGATTGA	TCTTTGACTGTGGGAGGATA	NM_011198.5	65	449
TNF-α	ATGAGCACAGAAAGCATGAT	TACAGGCTTGTCACTCGAAT	NM_013693.3	56	276
IL-6	TTCCATCCAGTTGCCTTCTT	ATTTCCACGATTTCCCAGAG	NM_031168.1	56	170

Il-1β	ATGGCAACTGTTCCTGAACT	CAGGACAGGTATAGATTCTT	NM_008361.3	56	563
MCP-1	TGATCCCAATGAGTAGGCTGG	ATGTCTGGACCCATTCCTTCT	NM_011333.3	56	132
GAPDH	AACGACCCCTTCATTGAC	TCCACGACATACTCAGCAC	NM_001289726.1	56	191

aP2: Adipocyte protein 2; C/EBPα: CCAAT/Enhancer binding protein α; AS: Fatty acid synthase; LPL: Lipoprotein lipase; PPARγ: Peroxisome proliferator-activated receptor γ; SREBP-1c: Sterol regulatory element binding protein 1c; iNOS: Inducible nitric oxide synthase; COX-2: Cyclooxygenase 2; TNF-α: Tumor necrosis factor α; IL: Interleukin; MCP-1: Monocyte chemoattractant protein 1; GAPDH: Glyceraldehyde 3-phosphate dehydrogenase

Disclaimer/Publisher's Note: The statements, opinions and data contained in all publications are solely those of the individual author(s) and contributor(s) and not of MDPI and/or the editor(s). MDPI and/or the editor(s) disclaim responsibility for any injury to people or property resulting from any ideas, methods, instructions or products referred to in the content.
